# 5-(4-Fluoro­phen­yl)-2-methyl-3-methyl­sulfinyl-1-benzofuran

**DOI:** 10.1107/S160053681103056X

**Published:** 2011-08-02

**Authors:** Pil Ja Seo, Hong Dae Choi, Byeng Wha Son, Uk Lee

**Affiliations:** a Department of Chemistry, Dongeui University, San 24 Kaya-dong Busanjin-gu, Busan 614-714, Republic of Korea; b Department of Chemistry, Pukyong National University, 599-1 Daeyeon 3-dong, Nam-gu, Busan 608-737, Republic of Korea

## Abstract

In the title compound, C_16_H_13_FO_2_S, the 4-fluoro­phenyl ring makes a dihedral angle of 38.75 (8)° with the mean plane of the benzofuran fragment. In the crystal, mol­ecules are linked by weak inter­molecular C—H⋯O hydrogen bonds.

## Related literature

For the pharmacological activity of benzofuran compounds, see: Aslam *et al.* (2009 ▶); Galal *et al.* (2009 ▶); Khan *et al.* (2005 ▶). For natural products with benzofuran rings, see: Akgul & Anil (2003 ▶); Soekamto *et al.* (2003 ▶). For structural studies of related 5-aryl-2-methyl-3-methyl­sulfinyl-1-benzofuran drivatives, see: Choi *et al.* (2006 ▶, 2009 ▶). For the synthesis of 2-methyl­benzofuran derivatives, see: Choi *et al.* (1999 ▶).
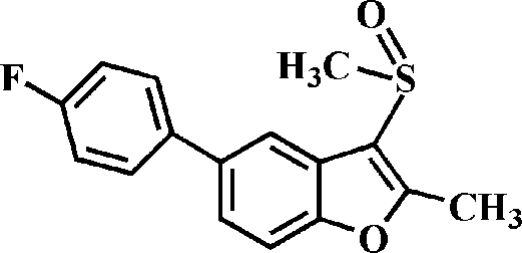


## Experimental

### 

#### Crystal data

C_16_H_13_FO_2_S
*M* *_r_* = 288.32
Monoclinic, 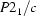

*a* = 15.101 (5) Å
*b* = 5.3150 (16) Å
*c* = 17.317 (5) Å
β = 93.606 (5)°
*V* = 1387.1 (7) Å^3^
*Z* = 4
Mo *K*α radiation
μ = 0.24 mm^−1^
*T* = 173 K
0.28 × 0.22 × 0.21 mm

#### Data collection

Bruker SMART APEXII CCD diffractometer
Absorption correction: multi-scan (*SADABS*; Bruker, 2009 ▶) *T*_min_ = 0.934, *T*_max_ = 0.952
13601 measured reflections
3478 independent reflections
2437 reflections with *I* > 2σ(*I*)
*R*_int_ = 0.055

#### Refinement

*R*[*F* ^2^ > 2σ(*F* ^2^)] = 0.049
*wR*(*F* ^2^) = 0.137
*S* = 1.05
3478 reflections
183 parameters
H-atom parameters constrained
Δρ_max_ = 0.32 e Å^−3^
Δρ_min_ = −0.22 e Å^−3^

### 

Data collection: *APEX2* (Bruker, 2009 ▶); cell refinement: *SAINT* (Bruker, 2009 ▶); data reduction: *SAINT*; program(s) used to solve structure: *SHELXS97* (Sheldrick, 2008 ▶); program(s) used to refine structure: *SHELXL97* (Sheldrick, 2008 ▶); molecular graphics: *ORTEP-3* (Farrugia, 1997 ▶) and *DIAMOND* (Brandenburg, 1998 ▶); software used to prepare material for publication: *SHELXL97*.

## Supplementary Material

Crystal structure: contains datablock(s) global, I. DOI: 10.1107/S160053681103056X/qk2019sup1.cif

Structure factors: contains datablock(s) I. DOI: 10.1107/S160053681103056X/qk2019Isup2.hkl

Supplementary material file. DOI: 10.1107/S160053681103056X/qk2019Isup3.cml

Additional supplementary materials:  crystallographic information; 3D view; checkCIF report

## Figures and Tables

**Table 1:** Hydrogen-bond geometry (Å, °)

*D*—H⋯*A*	*D*—H	H⋯*A*	*D*⋯*A*	*D*—H⋯*A*
C5—H5⋯O2^i^	0.95	2.36	3.245 (3)	156
C16—H16*B*⋯O2^ii^	0.98	2.43	3.319 (3)	151

Symmetry codes: (i) 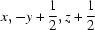; (ii) 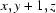.

**Fig. 1.**
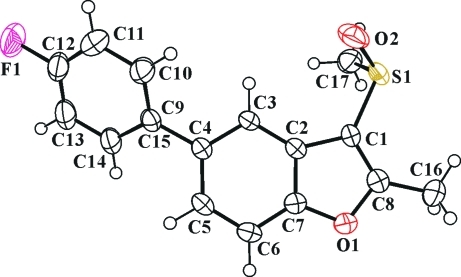
The molecular structure of the title compound with the atom numbering scheme. Displacement ellipsoids are drawn at the 50% probability level. H atoms are presented as a small spheres of arbitrary radius.

**Fig. 2.**
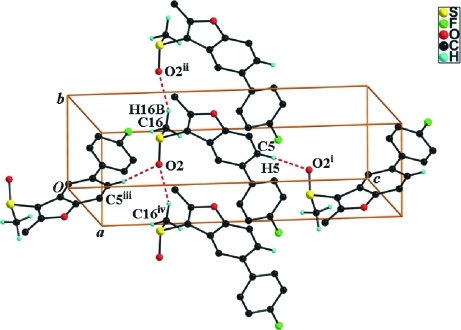
A view of the C—H···O interactions (dotted lines) in the crystal structure of the title compound. [Symmetry codes: (i) x, - y + 1/2, z + 1/2; (ii) x, y + 1, z; (iii) x, - y +1/2 , z - 1/2; (iv) x, y - 1, z.]

## supplementary crystallographic information

### Comment

Recently, many compounds having a benzofuran moiety have drawn much attention owing to their valuable pharmacological properties such as antibacterial and antifungal, antitumor and antiviral, and antimicrobial activities (Aslam *et al.*, 2009, Galal *et al.*, 2009, Khan *et al.*, 2005). These benzofuran derivatives occur in a wide range of natural products (Akgul & Anil, 2003; Soekamto *et al.*, 2003). As a part of our ongoing studies of the substituent effect on the solid state structures of 5-aryl-2-methyl-3-methylsulfinyl-1-benzofuran analogues, see: Choi *et al.* (2006, 2009), we report herein the crystal structure of the title compound.

In the title molecule (Fig. 1), the benzofuran unit is essentially planar, with a mean deviation of 0.011 (2) Å from the least-squares plane defined by the nine constituent atoms. The diheral angle formed by the 4-fluorophenyl ring and the mean plane of the benzofurn fragment is 38.75 (8)°. In the crystal packing (Fig. 2), molecules are linked by weak intermolecular C—H···O hydrogen bonds; the first one between a benzene H atom and the O atom of the sulfinyl group (Table 1; C5—H5···O2^i^), the second one between a methyl H atom of the methylsulfinyl group and the O atom of the sulfinyl group (Table 1; C16—H16B···O2^ii^).

### Experimental

5-(4-Fluorophenyl)-2-methyl-3-methylsulfanyl-1-benzofuran was obtained from 4'-fluoro-1,1'-biphenyl-4-ol and α-chloro-α-(methylsufanyl)acetone (Choi *et al.*, 1999). 77% 3-chloroperoxybenzoic acid (291 mg, 1.3 mmol) was added in small portions to a stirred solution of 5-(4-fluorophenyl)-2-methyl-3-methylsulfanyl-1-benzofuran (326 mg, 1.2 mmol) in dichloromethane (40 mL) at 273 K. After being stirred at room temperature for 4h, the mixture was washed with saturated sodium bicarbonate solution and the organic layer was separated, dried over magnesium sulfate, filtered and concentrated at reduced pressure. The residue was purified by column chromatography (hexane–ethyl acetate, 1:1 v/v) to afford the title compound as a colorless solid [yield 70%, m.p. 411–413 K; *R*_f_ = 0.46 (hexane–ethyl acetate, 1:1 v/v)]. Single crystals suitable for X-ray diffraction were prepared by slow evaporation of a solution of the title compound in ethyl acetate at room temperature.

### Refinement

All H atoms were positioned geometrically and refined using a riding model, with C—H = 0.95 Å for aryl and 0.98 Å for methyl H atoms. *U*_iso_(H)  = 1.2*U*_eq_(C) for aryl and 1.5*U*_eq_(C) for methyl H atoms.

### Crystal data

**Table:**

C_16_H_13_FO_2_S	*F*(000) = 600
*M**_r_* = 288.32	*D*_x_ = 1.381 Mg m^−^^3^
Monoclinic, *P*2_1_/*c*	Mo *K*α radiation, λ = 0.71073 Å
Hall symbol: -P 2ybc	Cell parameters from 2591 reflections
*a* = 15.101 (5) Å	θ = 2.6–26.4°
*b* = 5.3150 (16) Å	µ = 0.24 mm^−^^1^
*c* = 17.317 (5) Å	*T* = 173 K
β = 93.606 (5)°	Block, colourless
*V* = 1387.1 (7) Å^3^	0.28 × 0.22 × 0.21 mm
*Z* = 4	

### Data collection

**Table:**

Bruker SMART APEXII CCD diffractometer	3478 independent reflections
Radiation source: rotating anode	2437 reflections with *I* > 2σ(*I*)
graphite multilayer	*R*_int_ = 0.055
Detector resolution: 10.0 pixels mm^-1^	θ_max_ = 28.5°, θ_min_ = 1.4°
φ and ω scans	*h* = −20→20
Absorption correction: multi-scan (*SADABS*; Bruker, 2009)	*k* = −6→7
*T*_min_ = 0.934, *T*_max_ = 0.952	*l* = −22→22
13601 measured reflections	

### Refinement

**Table:**

Refinement on *F*^2^	Primary atom site location: structure-invariant direct methods
Least-squares matrix: full	Secondary atom site location: difference Fourier map
*R*[*F*^2^ > 2σ(*F*^2^)] = 0.049	Hydrogen site location: difference Fourier map
*wR*(*F*^2^) = 0.137	H-atom parameters constrained
*S* = 1.05	*w* = 1/[σ^2^(*F*_o_^2^) + (0.0651*P*)^2^ + 0.2344*P*] where *P* = (*F*_o_^2^ + 2*F*_c_^2^)/3
3478 reflections	(Δ/σ)_max_ < 0.001
183 parameters	Δρ_max_ = 0.32 e Å^−^^3^
0 restraints	Δρ_min_ = −0.22 e Å^−^^3^

### Special details

**Table:**

Geometry. All esds (except the esd in the dihedral angle between two l.s. planes) are estimated using the full covariance matrix. The cell esds are taken into account individually in the estimation of esds in distances, angles and torsion angles; correlations between esds in cell parameters are only used when they are defined by crystal symmetry. An approximate (isotropic) treatment of cell esds is used for estimating esds involving l.s. planes.
Refinement. Refinement of F^2^ against ALL reflections. The weighted R-factor wR and goodness of fit S are based on F^2^, conventional R-factors R are based on F, with F set to zero for negative F^2^. The threshold expression of F^2^ > 2sigma(F^2^) is used only for calculating R-factors(gt) etc. and is not relevant to the choice of reflections for refinement. R-factors based on F^2^ are statistically about twice as large as those based on F, and R- factors based on ALL data will be even larger.

### Fractional atomic coordinates and isotropic or equivalent isotropic displacement parameters (Å^2^)

**Table:**

	*x*	*y*	*z*	*U*_iso_*/*U*_eq_	
S1	0.17563 (4)	0.58042 (10)	0.28834 (3)	0.04103 (18)	
F1	0.55934 (10)	−0.3181 (3)	0.63856 (9)	0.0799 (5)	
O1	0.06789 (11)	0.7798 (3)	0.47995 (8)	0.0601 (5)	
O2	0.19330 (12)	0.3052 (3)	0.28237 (8)	0.0577 (5)	
C1	0.14479 (13)	0.6338 (4)	0.38282 (10)	0.0361 (4)	
C2	0.18189 (13)	0.5179 (4)	0.45385 (10)	0.0348 (4)	
C3	0.25025 (13)	0.3517 (3)	0.47389 (10)	0.0324 (4)	
H3	0.2843	0.2794	0.4353	0.039*	
C4	0.26806 (13)	0.2925 (4)	0.55191 (10)	0.0348 (4)	
C5	0.21503 (16)	0.3959 (4)	0.60763 (11)	0.0509 (6)	
H5	0.2269	0.3515	0.6604	0.061*	
C6	0.14668 (18)	0.5585 (5)	0.58852 (12)	0.0615 (7)	
H6	0.1112	0.6273	0.6267	0.074*	
C7	0.13179 (15)	0.6176 (4)	0.51116 (12)	0.0490 (6)	
C8	0.07767 (15)	0.7839 (4)	0.40150 (11)	0.0472 (5)	
C9	0.34346 (13)	0.1263 (4)	0.57592 (10)	0.0337 (4)	
C10	0.36519 (15)	−0.0773 (4)	0.53163 (13)	0.0471 (5)	
H10	0.3298	−0.1145	0.4858	0.056*	
C11	0.43708 (18)	−0.2285 (5)	0.55224 (15)	0.0636 (7)	
H11	0.4510	−0.3696	0.5216	0.076*	
C12	0.48785 (15)	−0.1697 (5)	0.61814 (13)	0.0506 (6)	
C13	0.46903 (15)	0.0255 (5)	0.66434 (13)	0.0518 (6)	
H13	0.5048	0.0598	0.7102	0.062*	
C14	0.39641 (16)	0.1737 (4)	0.64317 (12)	0.0499 (6)	
H14	0.3822	0.3112	0.6752	0.060*	
C15	0.01636 (16)	0.9524 (5)	0.35532 (14)	0.0584 (7)	
H15A	0.0304	1.1280	0.3682	0.088*	
H15B	−0.0449	0.9162	0.3672	0.088*	
H15C	0.0231	0.9241	0.3001	0.088*	
C16	0.28109 (15)	0.7332 (4)	0.29579 (13)	0.0502 (5)	
H16A	0.3181	0.6581	0.3382	0.075*	
H16B	0.2729	0.9127	0.3061	0.075*	
H16C	0.3102	0.7128	0.2472	0.075*	

### Atomic displacement parameters (Å^2^)

**Table:**

	*U*^11^	*U*^22^	*U*^33^	*U*^12^	*U*^13^	*U*^23^
S1	0.0546 (3)	0.0466 (3)	0.0211 (2)	0.0092 (2)	−0.00313 (19)	−0.0005 (2)
F1	0.0614 (9)	0.1015 (12)	0.0772 (11)	0.0432 (9)	0.0076 (8)	0.0292 (9)
O1	0.0642 (10)	0.0827 (12)	0.0348 (8)	0.0421 (9)	0.0126 (7)	0.0095 (8)
O2	0.0964 (13)	0.0440 (9)	0.0328 (8)	0.0079 (8)	0.0051 (8)	−0.0095 (6)
C1	0.0391 (10)	0.0436 (11)	0.0250 (9)	0.0089 (8)	−0.0030 (7)	0.0015 (8)
C2	0.0405 (10)	0.0402 (10)	0.0236 (9)	0.0068 (8)	0.0020 (7)	−0.0002 (8)
C3	0.0397 (10)	0.0355 (9)	0.0221 (8)	0.0055 (8)	0.0030 (7)	−0.0021 (7)
C4	0.0414 (11)	0.0377 (10)	0.0255 (9)	0.0067 (8)	0.0030 (8)	0.0034 (8)
C5	0.0663 (15)	0.0639 (14)	0.0234 (9)	0.0235 (12)	0.0089 (9)	0.0072 (9)
C6	0.0748 (17)	0.0814 (18)	0.0303 (11)	0.0408 (14)	0.0184 (11)	0.0079 (11)
C7	0.0547 (13)	0.0611 (14)	0.0318 (10)	0.0276 (11)	0.0077 (9)	0.0044 (9)
C8	0.0509 (13)	0.0587 (14)	0.0317 (10)	0.0169 (11)	0.0019 (9)	0.0042 (9)
C9	0.0413 (10)	0.0338 (9)	0.0263 (9)	0.0012 (8)	0.0033 (7)	0.0064 (7)
C10	0.0562 (13)	0.0401 (11)	0.0437 (12)	0.0099 (10)	−0.0058 (10)	−0.0051 (9)
C11	0.0757 (18)	0.0549 (15)	0.0602 (15)	0.0304 (13)	0.0033 (13)	−0.0061 (12)
C12	0.0417 (12)	0.0598 (14)	0.0506 (13)	0.0160 (10)	0.0065 (10)	0.0224 (11)
C13	0.0521 (13)	0.0600 (14)	0.0416 (12)	0.0059 (11)	−0.0100 (10)	0.0124 (11)
C14	0.0655 (15)	0.0508 (12)	0.0319 (11)	0.0152 (11)	−0.0088 (10)	0.0006 (9)
C15	0.0558 (14)	0.0726 (17)	0.0465 (13)	0.0303 (13)	0.0010 (11)	0.0111 (11)
C16	0.0527 (13)	0.0530 (13)	0.0458 (12)	0.0110 (11)	0.0107 (10)	0.0078 (10)

### Geometric parameters (Å, °)

**Table:**

S1—O2	1.4915 (17)	C8—C15	1.485 (3)
S1—C1	1.752 (2)	C9—C10	1.378 (3)
S1—C16	1.785 (2)	C9—C14	1.393 (3)
F1—C12	1.365 (2)	C10—C11	1.380 (3)
O1—C8	1.376 (2)	C10—H10	0.9500
O1—C7	1.379 (2)	C11—C12	1.370 (3)
C1—C8	1.345 (3)	C11—H11	0.9500
C1—C2	1.456 (2)	C12—C13	1.351 (3)
C2—C3	1.386 (3)	C13—C14	1.381 (3)
C2—C7	1.390 (3)	C13—H13	0.9500
C3—C4	1.397 (2)	C14—H14	0.9500
C3—H3	0.9500	C15—H15A	0.9800
C4—C5	1.404 (3)	C15—H15B	0.9800
C4—C9	1.480 (3)	C15—H15C	0.9800
C5—C6	1.371 (3)	C16—H16A	0.9800
C5—H5	0.9500	C16—H16B	0.9800
C6—C7	1.380 (3)	C16—H16C	0.9800
C6—H6	0.9500		
			
O2—S1—C1	106.43 (9)	C10—C9—C4	121.22 (17)
O2—S1—C16	106.77 (11)	C14—C9—C4	121.19 (18)
C1—S1—C16	98.46 (10)	C9—C10—C11	121.6 (2)
C8—O1—C7	106.32 (15)	C9—C10—H10	119.2
C8—C1—C2	107.68 (17)	C11—C10—H10	119.2
C8—C1—S1	124.69 (15)	C12—C11—C10	118.2 (2)
C2—C1—S1	127.56 (15)	C12—C11—H11	120.9
C3—C2—C7	119.61 (17)	C10—C11—H11	120.9
C3—C2—C1	136.24 (18)	C13—C12—F1	118.8 (2)
C7—C2—C1	104.13 (17)	C13—C12—C11	122.7 (2)
C2—C3—C4	118.74 (17)	F1—C12—C11	118.4 (2)
C2—C3—H3	120.6	C12—C13—C14	118.3 (2)
C4—C3—H3	120.6	C12—C13—H13	120.9
C3—C4—C5	119.56 (17)	C14—C13—H13	120.9
C3—C4—C9	120.27 (16)	C13—C14—C9	121.5 (2)
C5—C4—C9	120.16 (16)	C13—C14—H14	119.2
C6—C5—C4	122.28 (18)	C9—C14—H14	119.2
C6—C5—H5	118.9	C8—C15—H15A	109.5
C4—C5—H5	118.9	C8—C15—H15B	109.5
C5—C6—C7	116.87 (19)	H15A—C15—H15B	109.5
C5—C6—H6	121.6	C8—C15—H15C	109.5
C7—C6—H6	121.6	H15A—C15—H15C	109.5
O1—C7—C6	126.15 (19)	H15B—C15—H15C	109.5
O1—C7—C2	110.93 (17)	S1—C16—H16A	109.5
C6—C7—C2	122.92 (19)	S1—C16—H16B	109.5
C1—C8—O1	110.93 (17)	H16A—C16—H16B	109.5
C1—C8—C15	132.98 (19)	S1—C16—H16C	109.5
O1—C8—C15	116.06 (18)	H16A—C16—H16C	109.5
C10—C9—C14	117.57 (19)	H16B—C16—H16C	109.5
			
O2—S1—C1—C8	−137.1 (2)	C1—C2—C7—C6	−178.9 (2)
C16—S1—C1—C8	112.5 (2)	C2—C1—C8—O1	0.9 (3)
O2—S1—C1—C2	39.1 (2)	S1—C1—C8—O1	177.74 (16)
C16—S1—C1—C2	−71.2 (2)	C2—C1—C8—C15	178.8 (3)
C8—C1—C2—C3	−179.4 (2)	S1—C1—C8—C15	−4.3 (4)
S1—C1—C2—C3	3.8 (4)	C7—O1—C8—C1	−0.5 (3)
C8—C1—C2—C7	−0.9 (3)	C7—O1—C8—C15	−178.8 (2)
S1—C1—C2—C7	−177.67 (17)	C3—C4—C9—C10	−37.6 (3)
C7—C2—C3—C4	−1.3 (3)	C5—C4—C9—C10	143.6 (2)
C1—C2—C3—C4	177.1 (2)	C3—C4—C9—C14	140.8 (2)
C2—C3—C4—C5	1.9 (3)	C5—C4—C9—C14	−38.0 (3)
C2—C3—C4—C9	−176.79 (18)	C14—C9—C10—C11	−0.6 (3)
C3—C4—C5—C6	−1.3 (4)	C4—C9—C10—C11	177.9 (2)
C9—C4—C5—C6	177.4 (2)	C9—C10—C11—C12	−0.7 (4)
C4—C5—C6—C7	0.0 (4)	C10—C11—C12—C13	1.7 (4)
C8—O1—C7—C6	179.3 (3)	C10—C11—C12—F1	−179.7 (2)
C8—O1—C7—C2	−0.2 (3)	F1—C12—C13—C14	−179.8 (2)
C5—C6—C7—O1	−178.8 (2)	C11—C12—C13—C14	−1.2 (4)
C5—C6—C7—C2	0.7 (4)	C12—C13—C14—C9	−0.3 (4)
C3—C2—C7—O1	179.45 (19)	C10—C9—C14—C13	1.1 (3)
C1—C2—C7—O1	0.6 (3)	C4—C9—C14—C13	−177.4 (2)
C3—C2—C7—C6	−0.1 (4)		

### Hydrogen-bond geometry (Å, °)

**Table:**

*D*—H···*A*	*D*—H	H···*A*	*D*···*A*	*D*—H···*A*
C5—H5···O2^i^	0.95	2.36	3.245 (3)	156.
C16—H16B···O2^ii^	0.98	2.43	3.319 (3)	151.

Symmetry codes: (i) *x*, −*y*+1/2, *z*+1/2; (ii) *x*, *y*+1, *z*.
